# Comparison of the effects of preoperative melatonin or vitamin C administration on postoperative analgesia

**DOI:** 10.17305/bjbms.2019.4379

**Published:** 2020-02

**Authors:** Demet Laflı Tunay, Murat Türkeün Ilgınel, Hakkı Ünlügenç, Merthan Tunay, Feride Karacaer, Ebru Biricik

**Affiliations:** 1 Department of Anesthesiology, Faculty of Medicine, Balcalı Hospital, Cukurova University, Adana, Turkey; 2 Ministry of Health, Provincial Health Directorate, Adana, Turkey

**Keywords:** Melatonin, morphine consumption, postoperative analgesia, vitamin C

## Abstract

The analgesic benefit of melatonin and vitamin C as primary or adjuvant agents has been reported in various studies; however, their analgesic effects in the treatment of postoperative pain remain unclear. Thus, we aimed to evaluate the effect of single preoperative dose of oral melatonin or vitamin C administration on postoperative analgesia. In this study, we recruited 165 adult patients undergoing elective major abdominal surgery under general anesthesia. Patients were randomly divided into three equal (n = 55) groups. One hour before surgery, patients received orally melatonin (6 mg) in group M, vitamin C (2 g) in group C, or a placebo tablet in group P. Pain, sedation, patient satisfaction, total morphine consumption from a patient-controlled analgesia device, supplemental analgesic requirement, and the incidence of nausea and vomiting were recorded throughout 24 h after surgery. The mean pain score and total morphine consumption were found significantly lower in both M and C groups compared with group P (*p* < 0.001). There were no significant differences between group M and C with respect to pain scores (*p* = 0.117) and total morphine consumption (*p* = 0.090). Patients requested less supplemental analgesic and experienced less nausea and vomiting in groups M and C compared with group P. In conclusion, preoperative oral administration of 6 mg melatonin or 2 g vitamin C led to a reduction in pain scores, total morphine consumption, supplemental analgesic requirement, and the incidence of nausea and vomiting compared with placebo.

## INTRODUCTION

Effective postoperative pain management significantly improves patient satisfaction, reduces postoperative complications, and decreases the duration of hospital stay [1]. Opioids are widely chosen analgesics to treat postoperative pain; however, they have many possible adverse effects, such as nausea, vomiting, pruritus, and respiratory depression. Many techniques have been proposed to reduce opioid use such as preoperative, preventive or preemptive analgesia, and multimodal analgesia techniques [2,3].

Melatonin (N-acetyl-5-methoxytryptamine) is a hormone, principally produced by the pineal gland, and plays an important role in the circadian rhythm. It has chronobiotic, antioxidant, anxiolytic, analgesic, and sedative properties [4-6]. The analgesic benefit of melatonin in patients with acute and chronic pain syndromes has been shown greatly in both experimental and clinical studies [7].

Vitamin C (ascorbic acid) is one of the most powerful reductant agents with its antioxidant, neuroprotective, and neuromodulation effects [8-10]. Plasma vitamin C concentration has been reported to decrease after surgery due to increased oxidative stress [11]. In addition, it has been shown to reduce acute pain and the prevalence of complex regional pain syndromes with its antinociceptive effect [12-14].

Although clinical findings are inconsistent, some studies have demonstrated that the combination of an adjuvant and an opioid may diminish both opioid requirement for pain relief and the rate of opioid-related adverse effects [15]. Several multimodal approaches have been advocated to reduce opioid consumption based on different combinations of melatonin and vitamin C with patient-controlled analgesia (PCA), but the results regarding combination therapy success in perioperative settings are conflicting [16,17].

In this prospective, double-blind, randomized, controlled study, the preemptive (preventive) effect of melatonin and vitamin C, starting one hour before surgery, on postoperative analgesia, morphine consumption, sedation, patient satisfaction, and adverse effects were compared in patients undergoing elective major abdominal surgery. The hypothesis of this study was that preoperative oral melatonin and vitamin C administration would decrease postoperative morphine consumption and opioid-related adverse effects. Thus, the primary outcome measure was postoperative morphine consumption, and secondary outcomes were postoperative pain intensity, sedation, patient satisfaction, and opioid-related adverse effects in the first 24 hours postoperatively.

## MATERIALS AND METHODS

This study was approved by the Institutional Investigation and Ethics Committee (Approval number: TF2014LTP8) and conducted at Cukurova University in Turkey. The written informed patient consent was acquired from all participants. This study was also registered at ClinicalTrials.gov (Registration number: NCT02639741).

### Patients

One hundred sixty-five American Society of Anesthesiologists (ASA) status I-II adult patients aged between 18 and 65 years who underwent elective major abdominal surgery with general anesthesia were included in this prospective, double-blind, randomized, and controlled study. The exclusion criteria included a history of psychiatric disorders; chronic pain syndromes; mental impairment; drug or alcohol abuse; obstructive sleep apnea; severe asthma; chronic obstructive pulmonary disease; congestive heart, hepatic or renal failure; and patients with a history of an allergic reaction to the study drugs. Patients receiving drugs with known analgesic properties within 24 h before surgery were also excluded from the study. All patients were informed about the use of the PCA device and the visual analog scale (VAS) during the preoperative visit.

### Randomization and intervention

The patients were randomly allocated to one of three groups of 55, each using a computer-generated random number assignment. After assignment to interventions, the trial participants, care providers, and outcome assessors were all blinded. One hour before the start of surgery, patients in group M (n = 55) received 6 mg of melatonin (Melatonina 3 mg tb), patients in group C (n = 55) received 2 g of vitamin C (Solgar Vitamin C 1000 mg tb), and patients in group P (n = 55) received a placebo tablet, orally, in the preoperative unit. The tablets were given by an anesthetist who was not one of the observers, to ensure the double-blind design.

In the operation room, all patients were monitored using an electrocardiogram (ECG), non-invasive blood pressure (NIBP), peripheral oxygen saturation (SpO_2_), and end-tidal carbon dioxide (EtCO_2_). After anesthesia was induced with intravenous (IV) thiopental sodium (3–5 mg/kg), anesthesia maintenance was provided with 5–6% desflurane in a mixture of 60% nitrous oxide and 40% oxygen and remifentanil infusion (0.25–1 mcg/kg/min). Neuromuscular blockage was provided with IV rocuronium bromide (0.5 mg/kg).

Loading dose of 0.1 mg/kg IV morphine (Galen Co) and 75 mg intramuscular (im) diclofenac sodium (Voltaren, Novartis) was administered at the closure of the peritoneum for postoperative analgesia. Four milligrams of ondansetron hydrochloride (Zofer 4 mg/2 mL amp, Adeka, Istanbul) were administered after the closure of the abdomen for the prophylaxis of postoperative emesis.

Residual neuromuscular blockade was reversed with an IV neostigmine (0.05 mg/kg) and atropine (0.015 mg/kg) combination, prior to extubation.

Time to recovery was defined as the time period from extubation to the time to achieve an Aldrete score ≥ 9. Aldrete scores (judged by responding to commands, moving all extremities, breathing deeply and coughing freely, being fully awake or arousal on calling and oxygen saturation level >90% when breathing room air) [18] were evaluated by a blinded investigator at the end of surgery and recorded before patients were transferred to the postanesthesia care unit (PACU).

### Outcome and follow-up

Patients taken to the PACU after extubation were followed up in the unit for over 60 min, and a PCA device containing 0.3 mg/mL of morphine was inserted to the venous line and patients were allowed to use patient-controlled morphine. The settings for IV patient-controlled morphine were; bolus dose 0.2 mg/kg, with a lock-out time of 10 min and no continuous infusion. Whenever patients reported pain with a VAS score higher than 4 and asked for analgesic in addition to PCA therapy, 75 mg IM diclofenac sodium was also administered as supplement analgesia.

In the postoperative period, systolic and diastolic blood pressure (SBP and DBP), heart rate (HR), SpO_2_, pain (VAS), sedation and patient satisfaction scores, the consumed dose of PCA morphine, supplement dose of diclofenac sodium, and adverse effects (nausea, vomiting, pruritus, and allergic reactions) were recorded at 5, 10, 30, and 60 min in the PACU and 2, 4, 6, 8, 12, and 24 h postoperatively in the wards by blinded anesthetists.

Pain intensity was assessed using a 10-cm VAS ranging from 0 mm (no pain) to 10 cm (worst pain), sedation was assessed using a 6-point RAMSAY sedation scale (RSS) ranging from 1 to 6 (1 = anxious or restless, 2 = cooperative, oriented, 3 = responding to commands, 4 = brisk response to stimulus, 5 = sluggish response to stimulus, and 6 = no response to stimulus) [19], and patient satisfaction was evaluated using a 4-point numerical scale from 1 to 4 (1 = excellent satisfaction [VAS = 0], 2 = good satisfaction [VAS 1-2], 3 = dissatisfied [VAS 3-4], and 4 = very dissatisfied [VAS>5]) and these were all also recorded by blinded anesthetists. Patients were also assessed for severity of nausea/vomiting (n/v) using a numerical scale from 0 to 2 (0 = no nausea, 1 = mild n/v, and 2 = severe n/v).

### Sample size

In the calculation of sample size, 24 h PCA morphine consumption was targeted, thus the expected decrease in the 24 h PCA morphine consumption was defined based on the data from a previous study by Kanazi et al., which reported a 30% reduction of 24 h morphine consumption with the test intervention [20]. With an accepted power of 0.90 (β) and a significance level of 0.05 (α), the essential sample size would be 55 subjects for per arm (total 165 patients).

### Statistical analysis

The SPSS 20.0 package program was used to analyze the data. Categorical measurements are expressed as number and percentages (n[%]), numerical measurements are expressed as mean (95% confidence intervals [CI]) and standard deviation [SD] (if applicable, median, and minimum-maximum). Categorical measurements between groups were compared using the Chi-square test. Repeated measure analysis was used to assess change over time of numerical measurements at different time periods. The Kolmogorov–Smirnov test was used to determine whether continuous variables had a specific distribution. The level of statistical significance in all tests was considered as 0.05.

## RESULTS

**FIGURE 1:**
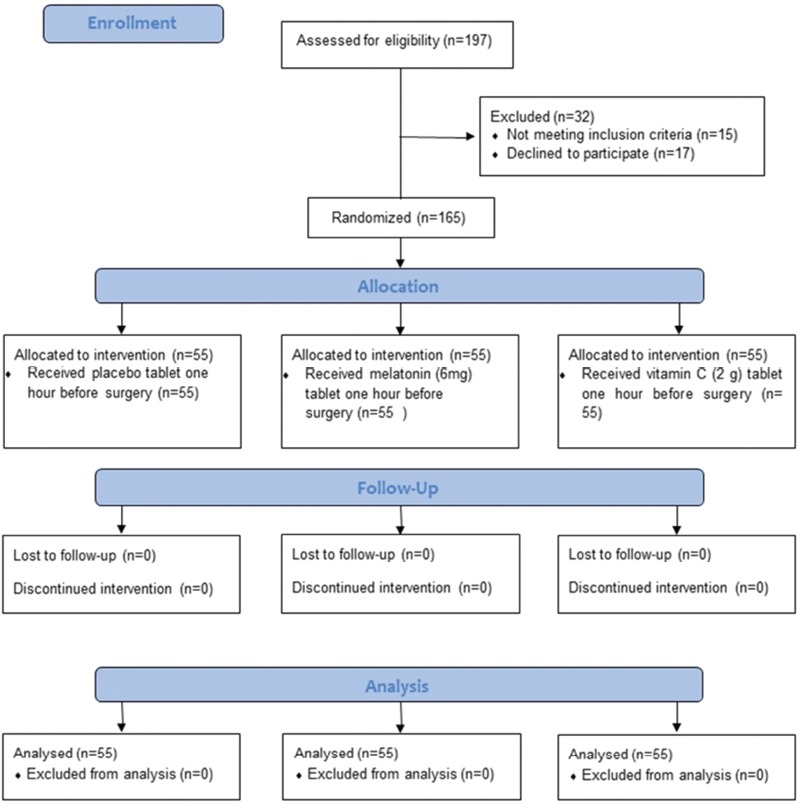
Flow chart describing enrollment, allocation, follow-up, and analysis of the study groups.

One hundred ninety-seven patients were evaluated for the study. Thirty-two patients were excluded from the study because they did not meet the inclusion criteria or refused to participate in the study. Thus, 165 patients were included in the study and were randomly allocated to receive either a single dose of melatonin (6 mg) or vitamin C (2 g) or placebo. There was no participant who discontinued or deviated from intervention protocols (Figure 1). The study was terminated when the number of 55 patients was achieved in each group by an anesthetist who assigned the participants to interventions.

There were no statistically significant differences between the groups in demographic variables and duration of surgery (Table 1). Time to recovery from anesthesia was found to be significantly longer in group M than in groups C and P [*p* = 0.019] (Table 1). Vital signs (SBP, DBP, HR, and SpO_2_) remained stable and there were no significant differences between the groups.

**TABLE 1:**
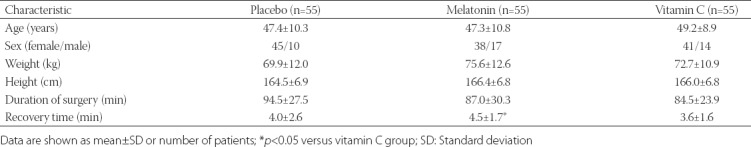
Demographic characteristics of patients, duratioAn of surgery, and recovery time

**FIGURE 2:**
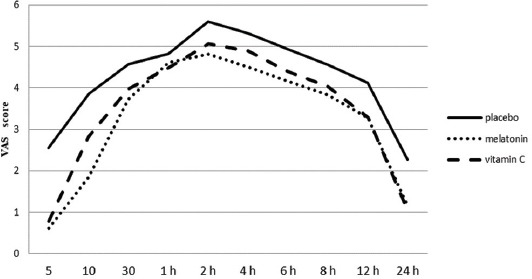
Visual analog scale (VAS) scores of patients during the first 24 hours postoperative period. VAS scores were significantly lower in group M and group C than in group P at all time points, except at one hour after surgery. Also, VAS scores increased for two hours in all groups and then decreased throughout the study period.

Postoperative VAS scores increased for two hours in all groups and then decreased throughout the study period (Figure 2). Postoperative VAS scores were significantly lower in group M and group C than in group P at all time points, except at one hour after surgery [*p* < 0.05] (Table 2).

**TABLE 2:**
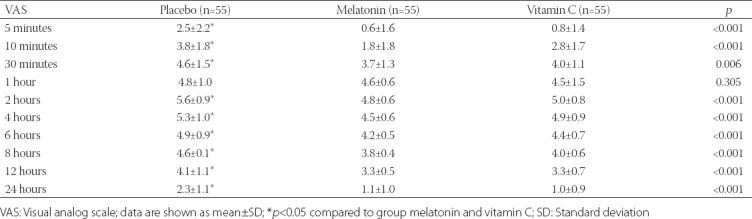
VAS scores of patients during the first 24 hours postoperative period

The mean postoperative patient-controlled morphine consumptions were 20.1 ± 6.8, 22.2 ± 6.5, and 24.6 ± 8.4 in group M, C, and P, respectively at 24 h and were found significantly lower in groups M and C than in group P [*p* = 0.007] (primary endpoint; Table 3). However, there was no significant difference between group M and C with respect to morphine consumptions at 24 h.

**TABLE 3:**
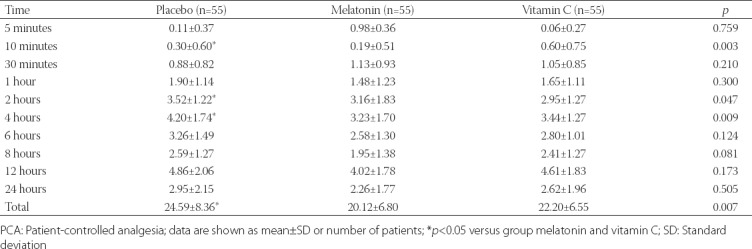
Postoperative PCA morphine consumption in groups

Patients requested less supplement diclofenac sodium in groups M and C compared with group P. The number and percentage of patients requiring supplement doses of diclofenac sodium were significantly lower in groups M and C than in group P (10.9%, 23.6%, and 41.8% in group M, C, and P, respectively; *p* = 0.001; Table 4). However, there was no significant difference between group M and C with respect to supplement doses of diclofenac.

**TABLE 4:**
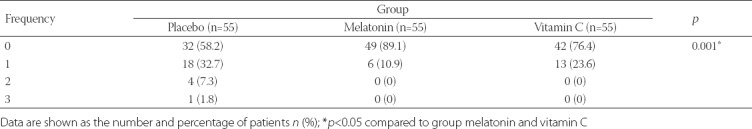
Number and percentage of the patients requiring supplement analgesic

Patient satisfaction scores were significantly lower in groups M and C at 24 h than in group P (percentages of patients having excellent or good satisfaction score at 24 h were 87%, 96%, and 64% in groups M, C, and P, respectively; *p* < 0.05).

Sedation scores were significantly higher in group M at 5, 10, and 30 min after surgery compared with groups C and P (*p* < 0.001). The number and percentage of patients having an RSS score of 3 at 5, 10, and 30 min in group M were 54 (98%), 44 (80%), and 25 (45%), respectively. The incidence of nausea and vomiting was significantly greater in group P at 4, 6, 8, 12, and 24 h postoperatively than in groups M and C (*p* < 0.05). Other adverse effects such as pruritus, hypotension, and bradycardia were minor, and there was no significant difference between the groups.

## DISCUSSION

In this prospective, double-blind, randomized, controlled study, both 6 mg melatonin and 2 g vitamin C, given one hour before the start of surgery, led to a reduction in VAS scores, total morphine consumption, supplement analgesic requirement, and the incidence of adverse effects compared with placebo.

Although several studies have reported the analgesic benefit of melatonin and vitamin C as primary or adjuvant agents, their synergistic or additive analgesic effects in the treatment of postoperative pain remain unclear [16,17]. The antinociceptive effects of both melatonin and vitamin C and their sites of action are not well understood; however, both agents may have an antioxidant function including inhibition of the formation of reactive oxygen and nitrogen species, which are involved in neuropathic pain, inflammatory pain, and tissue damage [8,21,22].

Some studies have shown that melatonin increases β-endorphin levels in the central nervous system [23,24] and interacts with opioid, γ-aminobutyric acid (GABA) or N-methyl-D-aspartate receptor system, centrally [24-26]. β-endorphin also has an antiinflammatory effect and probably plays a role in the peripheral analgesic effect [27]. The neuromodulation function of vitamin C, especially in dopamine- and glutamate-mediated neurotransmission through N-methyl-D-aspartate (NMDA) receptors, may also contribute to the effects of analgesia [10].

In an experimental study, the role of spinal melatonin receptors in the antinociceptive response was investigated using both mechanical and thermal stimuli, and melatonin was given through a lumbar spinal catheter. No analgesic effect was achieved with melatonin when administered alone, but an effective analgesic response was seen when combined with opioids [28]. In addition, both these agents can be safely administered with little or no adverse effects even at high doses [29,30]. In view of this evidence, the question arises as to whether melatonin or vitamin C have a morphine-sparing effect, reducing opioid-related adverse effects when used with morphine as an adjuvant agent.

In the present study, the morphine-sparing effect of the study drugs (melatonin and vitamin C) and/or synergistic interaction between morphine and the study drugs may be the underlying mechanism of the analgesic efficacy of the study drugs. This concern was reinforced by the lower VAS and morphine consumption, and patient satisfaction scores in groups M and C compared with group P. The highest VAS scores were achieved at postoperative two hours and these decreased gradually in all groups (Figure 2).

Melatonin and vitamin C have been recommended as adjuvants for the management of perioperative pain treatment [16,17]. However, the beneficial effect of adding vitamin C to opioids has been evaluated only in a few studies and the results are controversial. For instance, in a randomized placebo-controlled study by Kanazi et al., patients undergoing laparoscopic cholecystectomy receiving a 2-g single dose of oral vitamin C had lower morphine requirements compared with placebo [20]. A recent clinical study by Jeon et al. evaluated the effect of intraoperative IV vitamin C (50 mg/kg) infusion on morphine consumption and pain in patients undergoing laparoscopic colectomy. The authors showed that a high-dose infusion of vitamin C caused lower postoperative pain scores during the first 24 h after surgery and reduced morphine consumption in the early postoperative period [31]. Another recent prospective study by Jain et al. evaluating the postoperative long-term use of vitamin C (500 mg, twice a day for 6 weeks), showed that supplementation of vitamin C decreased analgesic requirements, improved VAS scores, and achieved better functional outcomes [32]. By contrast, Biswas et al. tested the effect of postoperative topical vitamin C drops, four times daily (3 mg) along with routine post-laser in situ keratomileusis medications for three days, on pain relief as evaluated using preset questionnaires on postoperative day 1 or 7, and they found no significant difference between the topical vitamin C and placebo groups [33].

The previous research regarding melatonin effects is also controversial. Borazan et al. reported that 6-mg oral tablets of melatonin, given one night and one hour before surgery, with patient-controlled tramadol, reduced intraoperative fentanyl consumption, decreased postoperative pain scores at 1, 2, 4, 6, 12, 18, and 24 h, and lowered postoperative patient-controlled tramadol consumption [34]. Similarly, Caumo et al. showed that 5-mg oral melatonin administered one night and one hour before surgery, significantly decreased postoperative pain scores and morphine consumption at 6, 12, 18, 24, 36, and 48 h [35]. In a recent study by Javaherforooshzadeh et al., the pain scores and need for opioid were lower throughout 24 hours postoperatively in patients receiving 6 mg melatonin 100 min before lumbar spine surgery, in comparison with a control group [36]. On the other hand, there have also been some contrary results, which failed to demonstrate any opioid-sparing effect or reduction in pain scores. In a study performed by Naguib and Samarkandi, patients received a single dose of oral 5-mg melatonin, 100 min before laparoscopic gynecologic surgery, and melatonin caused no decrease in intraoperative opioid consumption, postoperative pain score at 15, 30, 60, and 90 min, and postoperative analgesic consumption throughout a 90-min period postoperatively [37]. In another study, Acil et al. reported that a single dose of oral 5-mg melatonin given before laparoscopic surgery did not result in lower pain scores in the melatonin group [38]. The inconsistent results, in view of our study, may arise from the smaller sample sizes, short postoperative follow-up periods, and different types of surgery in the other studies.

In our study, less postoperative patient-controlled morphine consumption was achieved with the study drugs, and the patient-controlled morphine consumptions at 24 h were 20.1 ± 6.8 mg, 22.2 ± 6.5 mg, 24.6 ± 8.4 mg in group M, C and P, respectively, with the significantly lower values in M and C groups than P group (*p* < 0.007).

The plasma pharmacokinetics of vitamin C has been extensively studied, and the peak serum vitamin C level was observed at approximately four hours after oral administration [39,40]. Similarly, when melatonin has been administered as crystalline melatonin (80 mg in gelatin capsule) or as 3 mg orally, peak serum melatonin concentrations were reported within 60–150 min after its administration [41,42], and the effect persisted for up to 10–15 h [41-43]. However, some studies reported that melatonin was absorbed within 30 min after its administration and its elimination half-life was 45–60 min [41,44]. In our study, we observed a decrease in analgesia consumption approximately 240–270 min after vitamin C administration and 180–200 min after melatonin administration, which may correspond with the time when they reached their peak plasma levels.

The number and percentage of patients requiring a supplement dose of diclofenac were significantly lower in groups M and C than in group P (10.9%, 23.6%, and 41.8% in group M, C, and P, respectively; *p* = 0.001). However, there were no significant differences between groups M and C with respect to supplement doses of diclofenac (Table 4).

In the present study, the patients receiving oral melatonin had significantly higher sedation scores at 5, 10, and 30 min after surgery compared with groups C and P. This effect was attributed to the sedative effect of melatonin. The sedative effect of melatonin has been demonstrated in most previous studies [34,45]. Naguib and Samarkandi compared the sedative effects of 5-mg oral melatonin with 15-mg midazolam and found higher sedation levels with midazolam [37]. However, a similar difference between melatonin and midazolam was not found in the study performed by Acil et al. [38].

In our study, similarly, recovery time was also significantly longer in the melatonin group compared with the vitamin C and placebo groups (4.5 min, 3.6 min, 4 min in group M, C, and P, respectively). A similarly prolonged recovery time was also reported in the Borazan study where a melatonin group was compared with a placebo group [34].

We observed more intense nausea/vomiting in the placebo group only after two hours postoperatively, which corresponded with the time of significantly increased analgesic consumption in the placebo group compared with the melatonin and vitamin C groups.

The dose and administration route of melatonin and vitamin C were defined after examining different doses used in a variety of different clinical settings. The doses mostly ranged from 3 mg to 10 mg for melatonin [16] and 0.5 g to 3 g for vitamin C [17], so we chose 6 mg dose for melatonin and 2 g dose for vitamin C through an oral route. This is the first study in the literature to compare the postoperative analgesic effect of melatonin and vitamin C. However, a few studies that compared the antioxidant effects of melatonin with other antioxidant agents including vitamin C, showed that melatonin has a higher antioxidant effect than vitamin C and a combination of vitamin C and E [46-48].

There are some limitations to our study. First, we used diclofenac sodium for supplement analgesia when patients asked for additional analgesia. If we could have chosen an opioid analgesic as a rescue supplement analgesic it would be more convenient because of the faster onset time of analgesia. Second, we did not assess baseline and postoperative plasma concentrations of melatonin and vitamin C in the groups. Finally, we did not select patients undergoing any specific type surgery or separate patients by sex to compare sex-specific characteristics.

## CONCLUSION

In patients undergoing major abdominal surgery, oral administration of single-dose 6 mg melatonin and 2 g vitamin C, given one hour before surgery, decreased VAS scores, lowered PCA morphine consumption, reduced supplement analgesic requirement, and the intensity of nausea/vomiting compared with the placebo group. However, further studies investigating the dose and route of administration are needed to establish the optimal dosing regimen and route of administration.
